# The Cytoskeleton in Plant Immunity: Dynamics, Regulation, and Function

**DOI:** 10.3390/ijms232415553

**Published:** 2022-12-08

**Authors:** Jingyi Wang, Na Lian, Yue Zhang, Yi Man, Lulu Chen, Haobo Yang, Jinxing Lin, Yanping Jing

**Affiliations:** 1 National Engineering Research Center of Tree Breeding and Ecological Restoration, College of Biological Sciences and Technology, Beijing Forestry University, Beijing 100083, China; 2 Key Laboratory of Genetics and Breeding in Forest Trees and Ornamental Plants, Ministry of Education, Beijing Forestry University, Beijing 100083, China; 3 The Tree and Ornamental Plant Breeding and Biotechnology Laboratory of National Forestry and Grassland Administration, Beijing Forestry University, Beijing 100083, China

**Keywords:** actin filament, microtubule, plant immunity, dynamics, regulation, function

## Abstract

The plant cytoskeleton, consisting of actin filaments and microtubules, is a highly dynamic filamentous framework involved in plant growth, development, and stress responses. Recently, research has demonstrated that the plant cytoskeleton undergoes rapid remodeling upon sensing pathogen attacks, coordinating the formation of microdomain immune complexes, the dynamic and turnover of pattern-recognizing receptors (PRRs), the movement and aggregation of organelles, and the transportation of defense compounds, thus serving as an important platform for responding to pathogen infections. Meanwhile, pathogens produce effectors targeting the cytoskeleton to achieve pathogenicity. Recent findings have uncovered several cytoskeleton-associated proteins mediating cytoskeletal remodeling and defense signaling. Furthermore, the reorganization of the actin cytoskeleton is revealed to further feedback-regulate reactive oxygen species (ROS) production and trigger salicylic acid (SA) signaling, suggesting an extremely complex role of the cytoskeleton in plant immunity. Here, we describe recent advances in understanding the host cytoskeleton dynamics upon sensing pathogens and summarize the effectors that target the cytoskeleton. We highlight advances in the regulation of cytoskeletal remodeling associated with the defense response and assess the important function of the rearrangement of the cytoskeleton in the immune response. Finally, we propose suggestions for future research in this area.

## 1. Introduction

A large number of pathogenic microorganisms exist in the environment in which plants live. In order to counter the invasion by pathogens, plants have evolved two tiers of defense systems. Pattern-triggered immunity (PTI) is the first layer, generally activated by the perception of pathogen- or microbe-associated molecular patterns (PAMPs/MAMPs) and self-released damage-associated molecular patterns (DAMPs) via pattern-recognizing receptors (PRRs) [1,2,3]. This is often accompanied by an increased intracellular calcium concentration, bursts of reactive oxygen species (ROS), accretion of the lipid molecule, activation of mitogen-activated protein kinases (MAPKs), etc. These messengers trigger downstream reactions and fine-tune cellular signaling networks in plant immunity [3,4]. However, many pathogens have adapted to this mechanism and evolved effectors to bypass or straightly repress PTI [5]. Plants have also developed intracellular resistance proteins, i.e., nucleotide-binding leucine-rich repeat receptors (NLRs), that recognize bacterial effectors and usually cause a hypersensitive response (HR) to impede the pathogen spread, thus leading to effector-triggered immunity (ETI), which is the second layer of plant immunity [2,6].

The plant cytoskeleton, composed of microtubules and actin filaments, is a highly dynamic structure in cells and participates in many physiological activities, for instance, material transport, organelle movement, and cell division. In addition, the cytoskeleton also acts as a regulator of plant tolerance to biotic and abiotic stresses [7,8,9]. Microtubules are hollow tubular structures assembled from tubulin proteins, each composed of two globulin subunits that form a heterodimer. Meanwhile, actin filaments are composed of actin, usually forming a twisted chain about 7 nm in diameter. Microtubules and actin filaments are highly dynamic and undergo active polymerization and depolymerization processes, which are usually regulated by cytoskeleton-associated proteins, including microtubule-associated proteins (MAPs) and actin-binding proteins (ABPs), through nucleation of new filaments and polymerization, bundling, severing, and depolymerization of cytoskeletal fibers [7,10].

Recently, the cytoskeleton has been found to reorganize after pathogen attacks. This remodeling of the cytoskeleton is considered to serve as a platform for the perception and transduction of signals in the plant immune response, among which the function of actin filaments in plant immunity has been well-studied [9,11,12]. Meanwhile, the cytoskeleton also acts as the target of effectors produced by pathogens, making the interaction between host plants and pathogens more complex. Many ABPs have been identified to be necessary for actin cytoskeletal remodeling during the immune response [9,13,14,15]. Moreover, there are also some reports on the involvement of microtubules during the plant defense response [11]. In this review, we assess the recent progress in cytoskeletal remodeling during the immune response, sum up the known effectors acting on the cytoskeleton, and then focus, in particular, on the present understanding of the regulation of cytoskeleton dynamics in response to pathogens. We discuss the function of the cytoskeleton during plant immunity and finally propose some future prospects in this field.

## 2. Cytoskeletal Remodeling during Plant Immunity

The cytoskeleton undergoes dynamic remodeling upon sensing pathogens such as fungi, oomycetes, bacteria, and nematodes, and acts as a platform for immune signal perception and transduction. Meanwhile, some pathogens generate effectors that target the cytoskeleton to inhibit plant defense responses [9,11].

### 2.1. The Cytoskeleton Undergoes Remodeling during PTI

The increase in the density of actin filaments in epidermal pavement cells is a conserved feature of the PTI response [16]. The actin filament density increases in plants in response to fungal, oomycete, and bacterial infection, but the rearrangement of actin filaments varies with different types of pathogens. When plants respond to fungal and oomycete infections, actin filaments increase at the site where pathogens try to enter [17,18,19]. Pathogenic fungi can develop a unique structure named the appressorium during the invasion of plants, which can penetrate the periclinal wall of the host cells [20,21]. In a recent study, it was found that the actin filaments of *Arabidopsis* leaf epidermal cells change accordingly after being infected by the barley powdery mildew fungus *Blumeria graminis* f. sp*. hordei* (*Bgh*) [13]. In the early stage of infection, cortical actin filaments in leaf epidermal cells frequently accumulate around the invasion site with an increased density, forming concentrated, dome-like patches surrounding the penetration site. These actin patches appear to be less susceptible to treatment with the actin-depolymerizing drug latrunculin A (LatA), indicating that the filaments of actin patches at the penetration sites possess a higher stability [13]. In the late stage, polarized cytoplasmic actin filament bundles pass through the infected cell toward the site of penetration, displaying highly dynamic properties, and are gradually disintegrated at a later time [13]. The rearrangement of the actin cytoskeleton in epidermal cells during the infection of oomycetes is somewhat similar to that during the infection of fungi. Likewise, in the interaction of *Arabidopsis* with *Phytophthora sojae* and *Peronospora parasitica*, actin filaments reorganize to form large bundles at the site of oomycete infection [19]. This actin rearrangement after the perception of fungi and oomycetes facilitates the transport of defense compounds or proteins to pathogen attack sites, preventing pathogen invasion [13,14,22]. Genetic or chemical disruption of the actin cytoskeleton makes plants more sensitive to fungal pathogens and affects the accumulation of defense signaling molecules at the infection sites [23,24,25,26]. Unlike fungi and oomycetes, bacteria usually colonize on leaf surfaces or enter into the host mesophyll tissues through wounds or stomata and proliferate in this region [27,28]. Thus, the increase in actin filament density does not just exist locally. In *Arabidopsis* cotyledons inoculated with *Pseudomonas syringae* pv. *tomato* DC3000 (*Pst* DC3000) or the T3SS-deficient mutant *hrpH*, an elevated actin filament abundance was observed in the cortical cytoplasm of epidermal cells 6 h after inoculation. This early increase in actin filaments density is relevant to PTI [16]. In the case of bacterial pathogen perception, Henty-Ridilla et al. reported that disruption of the actin cytoskeleton with Latrunculin B (LatB) increased the *Arabidopsis* susceptibility to *Pst* DC3000 infection. However, there are also reports that LatB pretreatment enhanced *Arabidopsis* resistance to *Pst* DC3000 as well as *Brassica napus* resistance to *Leptosphaeria maculans*, reflecting the complexity of interactions between pathogenic bacteria and plant cytoskeleton [16,29]. Moreover, treatment of *Arabidopsis* leaves with both the flagellin peptide flg22 and the fungal cell wall component chitin can induce an increase in actin filament density as well. This process requires recognition of the signal by the corresponding receptor and downstream signal components, indicating that MAMP treatment is sufficient to cause changes in the actin arrangement in epidermal cells [16]. It was found that the treatment of hypocotyls with MAMPs induced alterations in the dynamics of individual actin filaments in epidermal cells [30,31]. In mock-treated cells, actin filaments can be divided into three types of origin: generated from the cytoplasm, generated at the ends of preexisting filaments, or generated at the sides of preexisting filaments. These three nucleation events are roughly equal in proportion. In conserved 26–amino acid peptide from bacterial elongation factor - (elf26) or chitin-treated cells, some kinetic parameters are changed. In elf26-treated cells, filaments tend to nucleate laterally, while in chitin-treated cells, they tend to nucleate terminally. In addition, the average length and lifetimes of actin filaments increase significantly, the frequency of filament–filament annealing increases, and severing decreases, but the elongation and regeneration rates do not change significantly [30,31]. As a result, the actin filament abundance in cells is increased. Hence, the increased density of actin filaments may be owing to changes in the dynamics of individual actin filaments.

In addition to epidermal pavement cells, stomata are also important gateways for pathogens to enter plants, which are often closed during plant immunity to prevent pathogens from entering. This process is called stomatal immunity, which is a part of plant innate immunity [32,33]. Stomatal movement is related to actin cytoskeleton dynamics. Actin filaments are arranged radially in open stomata, while in closed stomata, the actin cytoskeleton is mainly aligned longitudinally. Unlike in epidermal cells, *Pst* DC3000 infection does not change the abundance of actin filaments in *Arabidopsis* guard cells, but it affects the orientation of actin filaments. After sensing *Pst* DC3000, the actin filaments in stomata changes from a radial to a longitudinal arrangement, consistent with stomatal closure. However, the increase in actin filament density and bundling found in epidermal pavement cells is not observed during this process [34]. There are different reports on MAMP-induced actin rearrangement in guard cells. Shimono et al. found that both flg22 and chitin treatment induced stomatal closure, but the state of actin filaments was dominated by radial bundles. Meanwhile Zou et al. discovered that flg22 induced a decrease in the dynamicity of actin filaments, and the status of the actin filament distribution was randomly or longitudinally dominated. In both cases, no significant changes in actin filament density were detected [15,34]. The dynamics of the actin cytoskeleton in guard cells during the immune response as well as the involved regulation mechanisms still require further examination.

Microtubules also rearrange during pathogen infection, and disturbance of microtubules using chemical drugs or genetic methods increases plant sensitivity to pathogens, suggesting that changes in the microtubule cytoskeleton represent a portion of the plant defense response [9]. Interestingly, unlike the relatively consistent response of actin filaments in response to pathogens, the microtubule cytoskeleton appears to exhibit a variety of dynamic behaviors in different host–host interactions: some are aligned radially below the appressoria, some exhibit local microtubule depolymerization at the contact site of pathogens, and some show a circumferential arrangement [11]. For example, microtubules rarely aggregated or rearranged at the site of infection when *Arabidopsis* was attacked by *Bgh*, and no polar array of microtubules was observed in the later infection stage, suggesting that microtubules participate less in modulating focal cellular responses during the interaction between *Arabidopsis* and powdery mildew [13]. On the contrary, microtubules polarized at the infection site were observed in the interaction between barley and *Bgh*. In cells that the fungus succeeded in penetrating, microtubules loosened [35]. Hence, microtubules reorganize during the plant defense response, but the manner of rearrangement varies by plant and pathogen species. More studies are needed in the future to explore the detailed role of microtubules in plant immunity.

Besides the infection of pathogenic bacteria, nematode infections also affect the cytoskeletal organization of host plants. Root knot nematodes and cyst nematodes are the main nematodes that infect plants [11]. During infection, root knot nematodes form a multi-nucleated giant cell structure, usually consisting of six host cells, while cyst nematodes form syncytia [11,36]. In *Arabidopsis* syncytia, actin filaments and microtubules were found to be disrupted, and the mitotic apparatus was not observed. However, in giant cells, the mitotic apparatus was found, but the actin and cortical microtubules were disturbed. The depolymerization of the actin or microtubule cytoskeleton by chemical drugs causes nearly normal maturation of the infecting nematode, indicating that the disruption of the cytoskeleton is probably a necessity to allow nematodes to complete their life [37]. Thus, the cytoskeleton is also involved in the process of nematode infection.

### 2.2. Cytoskeletal Remodeling in ETI and Effectors Targeting the Cytoskeleton

**Figure 1:**
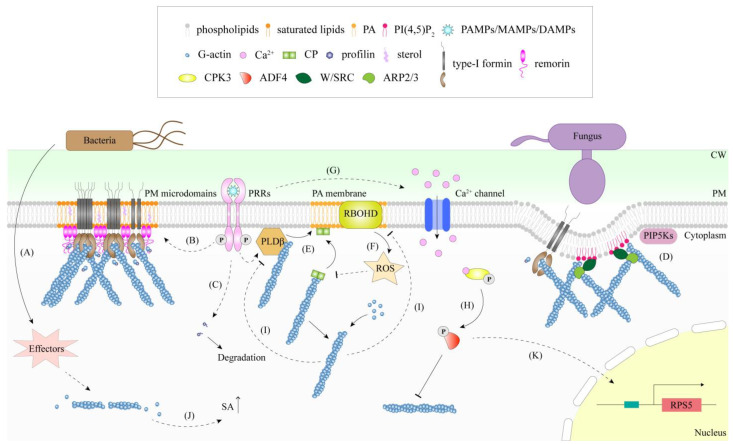
Regulation of actin filament dynamics in epidermal cells during plant immunity. (**A**) Sensing of bacteria induces actin filament remodeling, while bacteria produce effectors targeting the cytoskeleton. (**B**) When pathogens infect plants, PM-located PRRs are activated after sensing the corresponding PAMPs/MAMPs/DAMPs. PRRs activation induces the aggregation of type-I formin in membrane microdomains and promotes actin filament nucleation and assembly. (**C**) PRF3, which negatively regulates actin polymerization, is degraded after PRRs are activated. (**D**) During fungal infection, the ARP2/3 complex and type-I formin cooperate to form actin patches. (**E**,**F**) Upon sensing pathogens, PA levels are elevated through the activation of PLDβ, which further induces ROS production and inhibits CP activity. PLDβ binds F-actin and positively regulates its activity. (**G**) The activation of PRRs is often accompanied by an increased intracellular calcium concentration. (**H**) Calcium-dependent protein kinase 3 (CPK3) decodes calcium signal and phosphorylates ADF4 to separate it from actin filaments. (**I**) Changes in the actin filament density negatively regulate ROS production, probably through the modulation of PLDβ activity. (**J**) In addition, effectors released by bacteria break the actin cytoskeleton, which may improve plant resistance by increasing SA production. (**K**) Phosphorylated ADF4 is necessary for RPS5 expression, but the mechanism is still unclear. Solid lines indicate known or direct interactions, while dashed lines indicate unknown or indirect interactions. Arrows indicate the activation of signaling, while bars indicate an inhibitory effect. PRRs, pattern-recognizing receptors; PAMPs, pathogen-associated molecular patterns; MAMPs, microbe-associated molecular patterns; DAMPs, damage-associated molecular patterns. CW, cell wall; PM, plasma membrane.

The cytoskeleton also undergoes rearrangement during the ETI response. *Pst* DC3000 and not the T3SS-deficient mutant *hrpH* induces significant actin bundles 24 h after inoculation [16]. This illustrates that the formation of actin bundles in the late stage of bacterial infection is correlated with ETI. Researchers have found that many pathogens have evolved effectors that act on the cytoskeleton to enhance colonization (Figure 1A). Bacterial effectors that have been found to affect the cytoskeleton are listed in Table 1.

HopW1 is a bacterial effector from *Pst* DC3000 that targets the actin cytoskeleton. It interacts with AtACT7, reduces actin filamentous networks, and inhibits endocytosis. Expressing HopW1 in plants destroys the actin cytoskeleton, resulting in undetectable Lifeact-GFP-labeled actin filaments [38,39]. Another effector from *Pst* DC3000, HopG1, induces structural changes in actin filaments during infection. HopG1 was associated with actin bundling 24 h after infection. Loss of *hopG1* in *Pst* DC3000 results in reduced actin bundling and increased actin filaments in *Arabidopsis*. HopG1 disrupts actin stability and is associated with etiolation induction during infection [40]. Further studies have shown that HopG1 interacts with the mitochondrial-localized kinesin and is indirectly linked to actin through this interaction. Moreover, *Arabidopsis* kinesin mutants have reduced sensitivity to *Pst* DC3000 [40]. Previously, a kinesin-like calmodulin-binding protein was revealed to contain the MyTH4-FERM domain, allowing it to bind to both actin filaments and microtubules, suggesting that kinesin may dually bind microtubules and actin filaments [41]. Based on these results, it is hypothesized that HopG1-targeted kinesin is the mechanism that induces actin bundling during pathogen infection, thus inducing chlorosis and the formation of disease symptoms [40]. XopR, containing many disordered residues and potential plasma-membrane- and actin-binding motifs, is a newly identified effector of *Xanthomonas campestris*. During the early stage of infection, XopR goes through liquid–liquid phase separation (LLPS) via intrinsically disordered region (IDR)-mediated interactions, which recruits and condenses formin dimers into surface nanoclusters, promoting actin nucleation. In the late stage of infection, high concentrations of XopR cause formin to form large aggregates, inhibiting nucleation, promoting the formation of F-actin bundles, and reducing depolymerization by competing with actin depolymerizing factor (ADF) to bind actin [42].

In addition to targeting the actin cytoskeleton, many effectors have been identified to act upon microtubules in plant cells. Some of them directly destroy microtubule networks, and some target MAPs [43]. HopZ1a has acetyltransferase activity that interacts with and acetylates microtubules, leading to disruption of microtubule networks during late infection, interfering with secretion, affecting callose deposition, and blocking cell-wall-based defenses to enhance virulence [44]. HopE1 targets AtMAP65-1, a microtubule-associated protein that bundles microtubules, and dissociates it from the microtubule, disrupting the secretion of immune-related proteins. *Atmap65-1* mutants were more sensitive to *Pst* DC3000 and were deficient in the secretion of the immunity protein PR-1, suggesting that AtMAP65-1 plays a major part in plant immunity and protein secretion. However, the mechanism by which HopE1 dissociates MAP65-1 from microtubules and its effect on the microtubule network are still unclear and need to be further studied [45]. AvrBsT delivered into cells by *Pst* DC3000′s T3S system interacts with AtACIP1, which co-locates with microtubules in cells. AvrBsT infection induces the organization of large GFP-AtACIP1 aggregates dependent on an acetyltransferase activity, which is assumed to play a function in microtubule reorganization and microtubule-based processes [46]. The effector XopL is an E3 ubiquitin ligase, and its mutant lacking E3 ubiquitin ligase activity is selectively distributed to the microtubules. The filaments labeled by mutant XopL are usually aligned with stromules. Meanwhile, XopL inhibits stromule elongation and induces plastid aggregation. It is assumed that microtubules may be one of the targets of XopL. However, the precise effect of XopL on microtubules should be further investigated [47].

**Table 1:** Bacterial effectors targeting the cytoskeleton.

Pathogen	Effector	Activity	Target	Function	References
*Pseudomonas syringae*	HopW1	Unknown	Actin	Disruption of F-actin; inhibition of protein trafficking and endocytosis	[38,39]
*Pseudomonas syringae*	HopG1	Unknown	Mitochondrial- localized kinesin; indirectly associated with actin	Induction of actin bundling; induction of chlorosis	[40]
*Xanthomonas campestris*	XopR	Nucleation	Formin; actin	Activation of formin-mediated nucleation during early infection; promotes the formation of F-actin bundles and inhibits actin nucleation and depolymerization during late infection	[42]
*Pseudomonas syringae*	HopZ1a	Acetyltransferase	Tubulin	Acetylation and depolymerization of microtubules; inhibition of secretory	[44]
*Pseudomonas syringae*	HopE1	Unknown	AtMAP65-1	Dissociates AtMAP65-1 from microtubules; inhibits the secretion of immune-related proteins	[45]
*Xanthomonas euvesicatoria*	AvrBsT	Acetyltransferase	AtACIP1, which co-locates with microtubules	Acetylation of AtACIP1; induces the formation of AtACIP1 aggregates, which probably affect microtubule assembly	[46]
*Xanthomonas campestris*	XopL	E3 ubiquitin ligase	Unknown, probably microtubules	Inhibits stromule elongation; induces plastid aggregation	[47]

Nematodes also secrete effectors that destroy the cytoskeleton. It was found that MiPFN3 (*Meloidogyne incognita* Profilin 3), an effector secreted by root knot nematodes, can bind to actin monomers, disrupt actin polymerization, and reduce the filamentous actin network [36]. Manipulation of the cytoskeleton by nematodes may be a strategy to promote parasitism.

## 3. Regulation of Cytoskeleton Dynamics during Plant Immunity

As key regulators of cytoskeleton organization and dynamics, the activities of the cytoskeleton-associated proteins link the cytoskeletal remodeling to various pathogen infections. Several cytoskeleton-associated proteins have been revealed as vital intermediaries in the immune response and defense signaling [9].

### 3.1. Regulation of Cytoskeleton Dynamics by Cytoskeleton-Associated Proteins in Plant Immunity

At present, ABPs involved in actin cytoskeleton rearrangement in plant immunity have been sufficiently analyzed, including formin, profilin, actin-related protein (ARP2/3) complex, capping protein (CP), ADF, and villin. In contrast, MAPs involved in microtubule dynamic regulation in plant immunity remain to be further explored. Thus, we will mainly concentrate on the regulation of actin filaments by ABPs during the plant defense response.

Formin, an ABP responsible for regulating actin nucleation, can be grouped into two distinct phylogenetic clades: type-I and type-II formins [48]. The involvement of formin in plant immune responses, especially the dynamics and function of AtFH6, a type-I formin, has been well-studied. After sensing flg22, AtFH6 diffusion was limited, and it aggregated into nanoclusters to enhance actin nucleation [49]. Additionally, bacterial infection, flg22 and elf26 treatments also induced AtFH2 nanoclustering, suggesting that PAMP-triggered condensation may also be applicable to other type-I formins (Figure 1B) [49,50]. Another type-I formin, AtFH4, accumulates at the plasma membrane (PM), which fungi infect. AtFH4 contributes to fungal defense, and its complementation in *formin4/7/8* restores the fungus-sensitive phenotype of the mutant [22]. Moreover, the interaction between actin and cell wall appositions (CWAs) appears to be more direct in the *formin4/7/8* mutant lacking a finer-dispersed F-actin structure, suggesting that this localization of AtFH4 helps strengthen the distribution of local actin filaments [22].

Profilin interacts with formin through the polyproline motif of the formin FH1 domain, which then delivers the bound profilin-actin to the plus end for efficient actin polymerization [51,52]. Unlike the other profilin isoforms that promote polymerization in *Arabidopsis*, AtPRF3 inhibits actin polymerization and has been found to be involved in plant innate immune responses [53]. Upon flg22 treatment, the expression of *AtPRF3* decreased significantly, and the protein of AtPRF3 decreased 3 h after treatment (Figure 1C) but recovered 6 h later. The *Atprf3* mutant had a higher actin filament density than the wild type (WT), but both showed an increased actin filament density in response to flg22, indicating that AtPRF3 is not the only determinant of actin filament density during PTI. The assembly of actin filaments in *AtPRF3*-overexpressing plants is defective, which further illustrates the inhibitory effect of AtPRF3 on actin polymerization [53]. Furthermore, *Atprf3* was found to be more sensitive to *Pst* DC3000 than the WT. However, inoculation with the *Pst* DC3000 *hrcC* mutant, which only triggers PTI, can eliminate the higher sensitivity to bacteria, implying a role of AtPRF3 in ETI [53]. In cotton, the expression of *GhPFN2 (profilin)* was up-regulated after *Verticillium dahliae* infection. Plants overexpressing *GhPFN2* were more resistant to *V. dahliae* infection and exhibited a higher actin filament density and greater bundling [54]. Nevertheless, the mechanism of profilin’s involvement in actin remodeling, participating in plant immunity, requires further study.

In addition to the formin-profilin complex, ARP2/3 is another important nucleating complex that also functions in plant immunity [13,55]. The ARP2/3 complex has been shown to be involved in the formation of actin patches at fungal infection sites (Figure 1D). The density of actin filaments decreased in both *Atarp2-1* and *Atarp3-1* mutants, but actin bundles were more obvious in leaf epidermal cells. After inoculation with *Bgh*, *Atarp2-1* and *Atarp3-1* mutants had little or no actin patches below the penetration site and were more sensitive to infection [13]. Moreover, double mutants of the *ARP2/3* complex and *formin* showed higher penetration rates than the WT and single mutants, and actin patches formed below the *Bgh* penetration site were almost eliminated. The defects of the two nucleation systems also affect the accumulation of protein and defense materials, indicating that the two actin nucleation systems can act cooperatively to resist pathogen penetration [13]. Mutations in *AtARPC4* impair actin filament dynamics in the early stage of infection and enhance susceptibility to *Sclerotinia sclerotiorum* [56]. In addition to AtARPC4, other members of the ARP2/3 complex, such as AtARPC5 and AtARPC1a, are also required for *S. sclerotiorum* resistance, implying that a complete functional ARP2/3 complex is needed for disease resistance [56]. In tomato, ShARPC3 is associated with resistance to powdery mildew, and the transcription of *ShARPC3* is up-regulated after inoculation. Silencing of *ShARPC3* made tomatoes more susceptible to powdery mildew. On the contrary, overexpression of *ShARPC3* enhanced tomato powdery mildew resistance and rapidly induced HR and H_2_O_2_ production [55]. Similarly, wheat TaARPC3 and TaARPC5 are also involved in resistance to pathogens [57,58]. The function of ARP2/3 members in disease resistance is performed through the regulation of actin filament assembly during infection.

CP binds to the barbed ends of actin filaments and prevents the further polymerization of filamentous actin [9,10]. CP is negatively regulated during PTI, and its activity is inhibited. The actin filament density in *Atcpb-1* mutants did not change significantly after treatment with elf26 and chitin. Therefore, MAMP-induced actin filament dynamics were eliminated in *Atcpb-1* mutants [31]. *Atcpb-1* and *Atcpb-3* mutants affected callose deposition and transcriptional reprogramming and showed higher susceptibility to pathogens, while CP-overexpressed plants were more resistant to pathogens [31,59]. CP was revealed to be able to integrate signals from multiple PRR complexes and transform them into dynamic changes in the actin cytoskeleton [31].

ADFs are a family of depolymerizing and severing proteins, whose main function is to sever and depolymerize actin filaments. There are 11 ADF members in *Arabidopsis*, among which the role of AtADF4 in plant immunity has been well-studied [60,61]. The *Atadf4* mutation exhibited an altered actin cytoskeleton array in cells, resulting in a decrease in the frequency of severing and a remarkable increase in actin filament lengths and lifetimes [30,61]. This phenomenon is similar to the behavior of actin filaments in elf26-treated WT cells. Compared with the WT, the actin array in *Atadf4* was insensitive to elf26 treatment but displayed an increased actin filament abundance induced by chitin, indicating that AtADF4 may specifically play a role in elf26-mediated signaling pathway. However, another ADF mutant, *Atadf1*, was insensitive to both elf26 and chitin treatment, suggesting that AtADF1 appears to take part in the response to different MAMPs [30]. The effect of ADFs in plant immunity may vary with different ADF proteins. Some of them play a positive role in plant immunity. For instance, TaADF4 and TaADF7 positively regulate the immune response to *Puccinia striiformis* f. sp. *tritici* [62,63]. Some exhibit a negative regulatory function in plant immunity, such as GhADF6 and TaADF3 [64,65]. AtADF4 was also found to play a negative regulatory role in resistance to powdery mildew [66]. In addition to its role during PTI, AtADF4 also has important functions during ETI. *Atadf4* mutant were revealed to be more sensitive to *Pst* DC3000 AvrPphB, and the HR disappeared, but it restored the impaired resistance and HR activation after AtADF4 complementation [67]. ADFs act as either positive or negative regulators in plant immunity, but how these ADFs coordinate actin cytoskeleton dynamics and plant immune responses in vivo requires further research.

Villin is a member of the villin/gelsolin/fragmin superfamily and is a prominent regulator of actin [68]. It has a variety of activities in plants, including bundling, calcium-dependent severing, and plus-end-capping activities [68,69,70,71]. Currently, in the study of plant immunity, AtVLN3 is considered to be related to stomatal immunity but to not partake in MAMP-triggered actin remodeling of epidermal pavement cells [15]. In the guard cells of the *Atvln3* mutant, the actin array was more abundant but less bundled. The actin networks remained radially and randomly organized after flg22 treatment, which could not activate stomatal immunity to prevent bacterial entry, resulting in susceptibility to bacteria [15].

### 3.2. Regulation of Cytoskeleton-Associated Protein Activities during Plant Immunity

Plants recognize MAMPs through PRRs located in the plasma membrane when pathogens invade to activate the defense response [1,2,3]. Stimulation with several MAMPs, such as elf26, chitin, and flg22, induce cytoskeletal remodeling. Corresponding receptors have been identified in *Arabidopsis*. Elf26 and flg22 are sensed by the *Arabidopsis* pattern recognition receptor elongation factor-Tu (EF-Tu) receptor (AtEFR) and the *Arabidopsis* immune receptor FLAGELLIN SENSING2 (AtFLS2), respectively [72,73]. The *Arabidopsis* LysM-containing receptor-like kinases AtLYK1, AtLYK4, and AtLYK5 are responsible for chitin recognition [74,75,76]. It was found that the increased actin filament abundance after chitin treatment was impaired in *Atlyk* mutants. Similarly, treatment of *Atefr* mutants with elf26 did not cause an increase in the actin filament abundance. However, when *Atlyk* mutants were treated with elf26, or *Atefr* mutants were treated with chitin, the actin filament abundance increased [31]. Additionally, *Atfls2* mutants failed to increase the actin filament abundance in response to flg22 but could respond to chitin treatment to increase actin filaments [16]. These results indicate that the response of actin remodeling to various MAMPs requires an early signal event triggered by the corresponding PRR complex. In *Atbak1-4* and *Atbik1* mutants, the response to flg22 and chitin treatment failed to induce a significant increase in the actin filament abundance, suggesting that receptor-related signaling components also play a role in actin cytoskeletal remodeling [16]. Moreover, immune signal messengers such as phosphatidic acid (PA), ROS, and Ca^2+^ are known to act as upstream regulators of ABPs, modulating actin cytoskeletal remodeling during immune signal transduction. Furthermore, post-transcriptional modifications also fine-tune ABP activities in the process of plant immunity. In addition, it is also reported that ROP GTPase and nitric oxide (NO) signals are involved in regulating MAP activities or microtubule dynamics during the plant defense response.

The levels of PA, a signal phospholipid, are increased during plant cell immune signal transduction [9]. Exogenous PA can increase the abundance of actin filaments in plant cells [4]. Hydrolysis of structural lipids such as phosphatidylcholine by phospholipase D (PLD) is one of the ways in which PA is produced and functions in plant immunity and cytoskeletal remodeling [9]. There are 12 PLD isoforms in *Arabidopsis*, and among them, PLDβ is involved in the remodeling of the actin cytoskeleton in leaf epidermal cells in response to flg22 by producing PA and regulating downstream ROS production and CP activity (Figure 1E) [4]. The actin filament density in *Atpldβ1-2pldβ2* double mutants was not increased in response to multiple MAMPs and DAMPs treatments [4]. Furthermore, PA can bind CP, which inhibits the actin-binding activity of CP, allowing the elongation and contraction of actin filaments (Figure 1E) [77,78]. Inhibition of PLD-dependent PA production with the alcohol isomer 1-butanol reduced the actin filament abundance in *Arabidopsis* hypocotyl cells because CP activity could not be inhibited in the absence of PA. After elf26 or chitin sensing, 1-butanol treatment of WT *Arabidopsis* hypocotyl cells completely inhibited the actin response, while the actin filament abundance in *Atcpb-1* mutants was not affected by 1-butanol treatment, suggesting that plants negatively modulate CP through PLD/PA signaling during innate immunity [31]. In addition to PA, phosphatidylinositol-4,5- bisphosphate (PI (4,5) P_2_) also appears to play a role upstream of ABPs [13]. During powdery mildew attack, AtPIP5K2-YFP, which is accountable for the synthesis of PI (4,5)P_2_ in the plasma membrane, was significantly aggregated at the site where pathogens try to enter in the early stage of infection, and PI (4,5) P_2_ signaling was also focally accumulated, similar to the spatial and temporal pattern of actin patch formation [13]. Further studies revealed that locally aggregated PI (4,5) P_2_ is an upstream regulator of the WASP family verprolin homologous/SCAR regulatory complex (W/SRC)-ARP2/3 pathway that mediates the formation of actin patches (Figure 1D) [13]. The regulation of lipid signals in the cytoskeleton may be complex, involving the participation and interaction of many components, and there is still a great deal of research space to uncover the details.

ROS are often produced in the process of immune signal transmission. They serve as upstream signals of cytoskeletal remodeling in plant immunity and may also be regulated negatively by cytoskeletal feedback [4,59]. ROS in plant immunity are mediated by the NADPH oxidase RBOHD, which locates in the plasma membrane (Figure 1F) [79,80]. Loss of RBOHD eliminates the actin response to MAMPs and DAMPs, while H_2_O_2_ treatment of the WT and *AtrbohD* can increase the actin filament abundance [59]. H_2_O_2_ treatment was unable to induce actin remodeling in *Atcpb-1* cells, but it could restore the actin filament density increase in AtCP-OX (overexpressed) cells to a level similar to that of the WT [59]. These studies illustrate that CP is the mediator of ROS signaling to the actin cytoskeleton in the plant immune response (Figure 1F). As both PA and ROS act on CP to adjust actin dynamics and rearrangement, CP is considered a key node crosslinking different immune signaling pathways that regulate actin cytoskeletal remodeling.

The activation of calcium signal is involved in many signaling processes, including the plant immune response, which enable cells to quickly respond to the stimuli of the external environment (Figure 1G) [81]. Although the role of calcium in cytoskeletal remodeling during plant immunity has been rarely reported, it has a potential regulatory role in some ABPs during the defense response. AtPCaP1, also termed AtMDP25, locates in the PM and possesses actin cytoskeleton severing ability and microtubule destabilizing capacity [82,83]. AtPCaP1 participates in the oligogalacturonide- and flg22-induced defense response [84]. High concentrations of Ca^2+^ can induce the separation of AtPCaP1 from the plasma membrane. On the one hand, it binds and causes the instability of cortical microtubules [82]. On the other hand, it may directly interact with actin and sever actin filaments [83]. This provides a possible connection between defense-induced calcium signals and cytoskeletal remodeling in the immune process.

**Figure 2:**
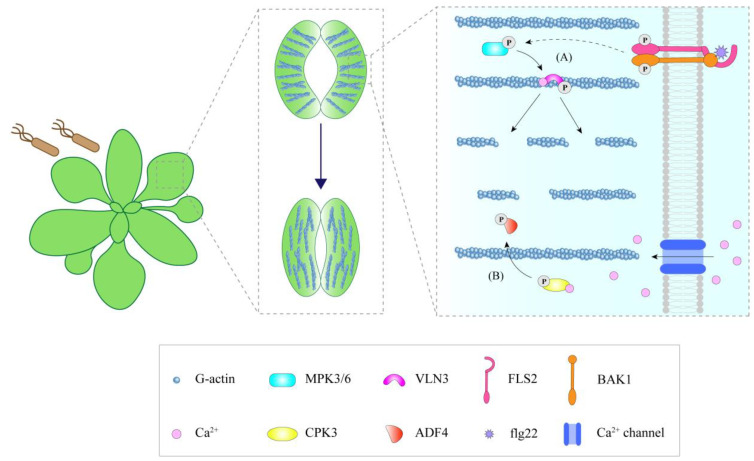
Regulation of actin filament dynamics during stomatal immunity. (**A**) After sensing pathogens, VLN3 in guard cells is phosphorylated through MPK3/6, which promotes the cleavage of actin filaments and causes the actin filaments to change from a radial to a longitudinal arrangement. (**B**) ADF4 can be phosphorylated by CPK3, and the activity of AtADF4 is inhibited, facilitating the actin reassembly process in stomatal immunity. Solid lines indicate known or direct interactions, while dashed lines indicate unknown or indirect interactions.

The phosphorylation status of ABPs affects their regulation activities in the actin cytoskeleton. During stomatal immunity, AtVLN3 is phosphorylated by MPK3/6 (mitogen-activated protein kinase 3/6) upon MAMP perception. This phosphorylation remarkably increases the activity of AtVLN3 to cut actin filaments, resulting in actin filament instability and the depolymerization of the radial actin array in the open stomata, thereby promoting actin recombination and initiating MAMP-induced stomatal closure (Figure 2A) [15]. Phosphorylation of AtADF4 affects its ability to bind actin filaments. Unphosphorylated AtADF4 can bind F-actin with high affinity, while phosphorylated AtADF4 has a low affinity for F-actin [85,86]. Upon flg22 treatment, calcium-dependent protein kinase 3 (CPK3) is induced, serving as a factor that phosphorylates AtADF4, separating it from actin filaments, preventing actin filaments from severing, and thus increasing the actin filament abundance in epidermal pavement cells during immunity (Figure 1H) [86]. Beside its role in epidermal cells, AtADF4 phosphorylation by AtCPK3 may be involved in stomatal immunity, linking immune-induced alterations in actin filament organization to the regulation of stomatal closure (Figure 2B) [86]. In addition, AtFH6 is also phosphorylated in response to flg22 [87]. However, whether this phosphorylation is involved in the accumulation of formin during PTI as well as its effect on actin organization requires further study.

In terms of microtubules, although the signaling pathways involved in microtubules in plant immunity are still unclear, some progress has been made in this area. In the research of barley, it was found that microtubules are regulated by ROP proteins. Cell perception of *Bgh* can lead to the activation of two ROP proteins, HvRACB and HvRAC1, in barley, further enhancing the activity of HvRBK1 and recruiting it to the cell cortex. HvRBK1 may support microtubule stability, and *HvRBK1* knockout causes microtubule depolymerization and increases susceptibility to *Bgh* infiltration [88]. HvRACB also binds to HvMAGAP1, which binds microtubules and is involved in the penetration resistance to *Bgh* [35]. In addition, pathogen attack was also reported to induce the generation of NO signals [89]. In *Arabidopsis* leaves, *Verticillium dahliae* (VD) toxins caused the production of NO and the disruption of cortical microtubules. Further analysis indicated that the elimination of NO during VD-toxins treatments reduced the destabilization of microtubules, suggesting that NO can serve as an upstream signal to induce the disaggregation of microtubules [90]. However, the mechanism by which NO acts on microtubules needs to be studied further.

## 4. Cytoskeleton Function in Plant Immunity

### 4.1. Cytoskeleton and Membrane Microdomain Interplay Forming Plant Immunity Platforms

Besides the cytoskeleton, membrane microdomains could also provide a platform for sensing plant immune signals [91]. Membrane microdomains are lipid-order domains enriched in sphingolipids and cholesterol, which contain marker proteins such as hypersensitive induced reaction (HIR) proteins, remorins (REMs), and flotillins, which label different microdomains [92]. As both serve as platforms for immunosensing, the cytoskeleton and microdomains interplay during plant immunity. On the one hand, microdomains are dynamically reorganized during plant defense against pathogens, while the cytoskeleton is found to be able to modulate the arrangement of microdomain. Combined cytoskeleton inhibitor treatment and proteomics analysis indicated that microtubules govern the size and density of membrane microdomains [93]. In *Arabidopsis*, the cytoskeleton, especially the microtubules, confines the dynamics of the microdomain marker protein AtHIR1, promoting the oligomerization of AtHIR1, hence advancing the formation of the AtHIR1-associated immune complex upon the perception of pathogens [94]. On the other hand, microdomain marker remorin proteins recruit and promote the nanoclustering of AtFH6 upon PTI activation, thereby enhancing the nucleation of actin filaments in a time-dependent manner and triggering actin cytoskeletal remodeling (Figure 1B) [50]. Hence, microdomains mediate the condensation of ABPs and subsequently regulate the assembly and dynamics of actin.

### 4.2. The Cytoskeleton Regulates the Dynamics and Turnover of PRRs

The dynamics of membrane proteins are usually considered to be closely related to their functions and the signal transduction they are involved in. Previously, the cytoskeleton has been revealed to participate in the regulation of PRR dynamics. The pathogen receptor AtFLS2 in both *Arabidopsis* protoplast and epidermal cells exhibited reduced lateral displacement after sensing flg22 ligands [95]. Further research demonstrated that flg22 induces the clustering of the AtFLS2 receptor in *Arabidopsis* leaf epidermal cells [96]. Meanwhile, depolymerization of either actin or microtubule cytoskeletons resulted in increased lateral diffusion of AtFLS2 [97], implying a confined effect of the cytoskeleton on the lateral movement of PRR, which is considered to be facilitated for the receptor clustering upon sensing pathogens.

During the plant immune response, many receptors undergo endocytosis after sensing the related ligands. For example, AtFLS2 and AtPep peptides receptor 1/2 (AtPEPR1/2) are internalized after being activated by flg22 and plant elicitor peptide 1 (pep1), respectively [96,98]. After endocytosis, some receptors are delivered to the vacuole and degraded there, which is thought to be a means for signal attenuation. Some receptors are recycled back to the plasma membrane, considered as a part of the signal transduction [99]. Myosin is an actin-based cytoskeletal motor protein, mediating the transport of materials along actin filaments through ATP hydrolysis [100]. Notably, the myosin inhibitor 2,3-Butanedione monoxime (BDM) inhibits the endocytosis of AtFLS2, indicating that the actin-myosin system plays a major role in flg22-induced endocytosis [101]. Given the important role of the spatiotemporal dynamics of receptors and receptor-mediated endocytosis as well as the function of the cytoskeleton in regulating PRR dynamics in PM and PRR endocytosis, this illustrates the significant role of the cytoskeleton during the immune response.

### 4.3. The Cytoskeleton Provides Anchor Site or Track for Host Organelles

Increasing data suggest that the host organelles play a crucial role in immune regulation, depending on the involvement of the cytoskeleton. Many host organelles have been found to accumulate at the pathogen infection site, which is thought to intensify the immune response. During *Bgh* infection, mitochondria were observed to move in a linear orbit and aggregate at the site of penetration. Similarly, movement of Golgi and peroxisomes toward the site of infection was observed [18]. Chloroplasts accumulate at the pathogen infection interface during *Phytophthora infestans* infection. Chemical disruption of actin polymerization significantly reduced chloroplast accumulation at the pathogen interface [102], indicating that the aggregation of chloroplasts requires the participation of the actin cytoskeleton. Besides chloroplasts, actin patch assembly was observed in coordination with the endoplasmic reticulum dynamics around the site of pathogen penetration during the early stages of *Bgh* penetration [13]. In *Arabidopsis* epidermal cells, along with actin filaments concentrating at the oomycete infection site, the endoplasmic reticulum and Golgi also aggregate there, suggesting the active production and secretion of immune compounds around this site [19]. The inhibition of myosin activity suppressed the long-distance movement of organelles in *Arabidopsis* leaves, implying that host organelle transport upon pathogen attack depends on the actomyosin system [18]. Apart from providing tracks for organelle movement during plant immunity, the cytoskeleton also provides an anchor site for organelles. When plants defend against pathogenic microorganisms, the chloroplasts form stromules that connect to the nucleus to transmit signaling molecules and defense proteins. Stromules extend along microtubules, and actin filaments provide anchor sites for stromules that guide chloroplast aggregation around the nucleus during innate immunity [103,104]. Overall, the cytoskeleton is involved in the repositioning of host organelles, which is relevant to the immune response intensities.

### 4.4. The Cytoskeleton Modulate the Deposition of Defense Compounds and Proteins

As an intracellular transport track, the cytoskeleton is related to the rapid relocalization of immune components to the infection site [105]. Callose deposits are important for strengthening cell walls and preventing pathogen invasion. This process requires the involvement of the cytoskeleton. Defects in actin filaments and microtubules cause a loss of callose deposits in infected plants [30,31,44,45,106]. In addition, callose deposition also requires myosin XI, an actin-based motor protein [18]. The *Arabidopsis* penetration resistance 3 (AtPEN3) is involved in non-host resistance to pathogens. Local accumulation of AtPEN3 is induced by the perception of PAMPs and requires actin filaments. Depolymerization of the actin cytoskeleton with either cytochalasin E or LatB reduces AtPEN3 accumulation at the site of pathogen penetration. However, local accumulation of AtPEN3 does not require microtubules [107]. Moreover, actin filaments are also involved in the transport of DAMPs to send signals to neighboring cells to activate defensive responses [14,20].

### 4.5. The Cytoskeleton Is Involved in Immune Signaling Regulation

The cytoskeleton is closely related to the production of immune signals. ROS production is a feature of innate immune responses, and changes in cytoskeletal dynamics have been reported to affect this process. Disruption of the actin cytoskeleton by LatB enhanced the ROS burst of *Arabidopsis* after flg22 treatment [59]. Mutations and overexpression of ABPs, such as AtPRF3 and CP, resulted in a more significant increase in ROS production after flg22 perception, implying a correlation between actin status and ROS generation [53,59]. In the absence of MAMPs, LatB treatment does not trigger ROS production, indicating that actin remodeling plays a significant role in ROS induction during the immune response [59]. In addition to the actin cytoskeleton, microtubules also act in flg22-induced ROS bursts. Mutants with impaired microtubule dynamics showed decreased ROS production after flg22 treatment, reflecting the divergent roles the actin and microtubule cytoskeletons play in the induction of ROS in the plant defense response [45,46]. Thus far, we know that the cytoskeleton participates in ROS production in PTI, but the underlying mechanism is still unclear. Previously, the CP protein was reported to transmit ROS signals to the actin cytoskeleton during the plant immune response, while ROS production induced by flg22 in *Atpldβ1-2pldβ2cpb-1* and *Atpldβ1-1cpb-1* mutants was much higher than that in the WT or *Atcpb-1* single mutants. This is probably because there is a feedback loop from the actin cytoskeleton to ROS production (Figure 1I). Notably, the polymerization of *Arabidopsis* and tobacco pollen tube actin has been reported to be able to activate PLDβ; therefore, the feedback regulation of actin in ROS production is probably achieved through the modulation of PLDβ activity (Figure 1I) [4,59,108,109].

Salicylic acid (SA) is another plant immune signal produced during pathogen infection. Researchers have found that actin depolymerization increases the expression level of SA marker genes and induces SA synthesis (Figure 1J) [29,110]. Treatment of *Arabidopsis* seedlings with 200 nM LatB for 24 h increased SA levels by seven times. Further analysis indicated that the gene expression of isochorismate synthase (ICS), which is responsible for SA synthesis, was induced by LatB treatment, suggesting that drug-induced actin depolymerization stimulates an ICS-dependent pathway that is responsible for SA biosynthesis during this process [29]. Moreover, *Arabidopsis* seedlings pretreated with LatB showed stronger resistance to the bacterial pathogen *Pst* DC3000, and *Brassica napus* pretreated with LatB displayed an induced SA pathway and improved resistance to *Leptosphaeria maculans* [29]. In contrast to actin, only a limited effect on SA marker genes was observed after depolymerization of microtubules with oryzalin, which was much lower than that after actin depolymerization [110]. In addition, *Arabidopsis map65-3* mutants have about 2.5-fold more SA compared with the WT. Upon *Hyaloperonospora arabidopsidis* infection, SA levels in *map65-3* mutant increased more than 10-fold compared to the WT, and the *map65-3* mutants showed dramatically reduced infection [111]. Overall, both actin and microtubule dynamics during the plant defense response are connected with SA signaling, in which the actin cytoskeleton is more closely linked to the SA pathway. The components responsible for triggering SA signaling via the actin state upon pathogen attack still need to be identified.

The cytoskeletal remodeling upon pathogen attack finally influences the expression of defense-related genes, triggering the relevant immune response. For example, in *Arabidopsis adf4* mutants showing an altered actin cytoskeleton, the expression of the resistance protein *AtRPS5* was significantly reduced. Further studies revealed that AtADF4 phosphorylation, which affects its cytoskeletal localization, is necessary for *AtRPS5* expression (Figure 1K) [85]. For microtubules, pharmacological treatment of microtubules triggered the expression of defense genes [112], indicating a close relevance between cytoskeleton status and defense-related gene expression.

## 5. Conclusion and Future Perspective

Overall, the cytoskeleton is implicated in plant immune responses. The density of actin filaments in epidermal cells increases when plants are invaded by fungi, oomycetes, and bacteria. Unlike bacteria, actin filaments focus on the penetration site when dealing with fungi and oomycetes, while bacteria evolve effectors targeting the cytoskeleton. In guard cells, the pattern of microfilament rearrangement influences the opening and closing of stomata. Several ABPs have been revealed to fine-tune the actin cytoskeleton dynamics, linking actin remodeling to pathogen infections. Microtubules also play a role in plant immunity, but their changes are different among different species. The reorganization of the cytoskeleton will eventually promote the transportation and accumulation of defense proteins, antibacterial compounds, and cell wall components at the infected sites of pathogens, coordinate the movement of organelles, and cause variation in resistance. The damaged cytoskeleton may be sensed by an unknown mechanism, further causing downstream signal changes. Moreover, some ABPs not only participate in the regulation of actin dynamics during PTI but also participate in the process of ETI and regulate the expression of resistance protein genes, which makes the signal network of the cytoskeleton in plant immunity more complicated.

Recent studies have enhanced our knowledge of the function of the cytoskeleton in plant immunity, but there are still many problems to be further studied: (1) How is the signal transmitted to actin rearrangement after sensing pathogens, and how does the mechanism of sensing the cytoskeleton status to trigger a downstream defense response? The exact signal transduction pathway and the components involved need to be clarified. (2) PTI and ETI do not work alone but are interrelated. ABPs connecting PTI and ETI need to be excavated, and the mechanism underlying the synergetic regulation of the actin cytoskeleton in both PTI and ETI needs to be further explored. (3) It has been found that fungi also have effectors [113], so will the cytoskeleton interact with fungal effectors in the process of fungal infection? More research is needed to reveal these details. (4) What is the exact role of the microtubule cytoskeleton and the involved regulation mechanism in the plant immune response? It might be interesting to investigate the component targeting both microtubules and actin filaments and coordinating both cytoskeletons during plant immunity. (5) It is preferable to figure out whether the cytoskeleton function in plant immunity is universal among plants and whether the mechanism involved is conservative. (6) As a complex dynamic filamentous network throughout the whole cell, it would be interesting to reveal the real-time dynamic changes in the cytoskeleton and the ultrastructural characterization of pathogen and host responses during the interaction. The recent application of high-spatiotemporal-resolution imaging techniques and volume imaging systems [114,115,116] will help us better understand the cytoskeleton remodeling and function during the plant defense response. Recently, Vernet et al. successfully observed the nematode–root interactions in tomato using light sheet fluorescence microscopy (LSFM) and optical projection tomography (OPT) [117]. Moreover, simultaneous imaging of *S. meliloti* (magenta) and the host cytoskeleton in cytoskeletal marker gene transgenic alfalfa plants was realized by using light-sheet and super-resolution microscopy, providing an approach to track the dynamic changes in the host cytoskeleton and pathogenic microbes over a long time at the super-resolution level [118].

## Data Availability

Not applicable.

## Abbreviations

ABPs	Actin-binding proteins
ADF	Actin depolymerizing factor
ARP2/3	Actin-related protein 2/3
BDM	Butanedione monoxime
*Bgh*	*Blumeria graminis* f. sp*. hordei*
CP	Capping protein
CPK3	Calcium-dependent protein kinase 3
CW	Cell wall
CWAs	Cell wall appositions
DAMPs	Damage-associated molecular patterns
EF-Tu	Elongation factor-Tu
EFR	Elongation factor-Tu receptor
elf26	Conserved 26-amino acid peptide from bacterial elongation factor
ETI	Effector-triggered immunity
flg22	Flagellin 22
FLS2	Flagellin sensing 2
HIR	Hypersensitive induced reaction
HR	Hypersensitive response
ICS	Isochorismate synthase
IDR	Intrinsically disordered region
LatA	Latrunculin A
LatB	Latrunculin B
LLPS	Liquid-liquid phase separation
LSFM	Light sheet fluorescence microscopy
LYK	LysM-containing receptor-like kinase
MAMPs	Microbe-associated molecular patterns
MAPKs	Mitogen-activated protein kinases
MAPs	Microtubule-associated proteins
MiPFN3	Meloidogyne incognita profilin 3
MPK3/6	Mitogen-activated protein kinase 3/6
NLRs	Nucleotide-binding leucine-rich repeat receptors
NO	Nitric oxide
OPT	Optical projection tomography
OX	Overexpressed
PA	Phatidic acid
PAMPs	Pathogen-associated molecular patterns
PEN3	Penetration resistance 3
pep1	Plant elicitor peptide 1
PEPR1/2	Pep peptides receptor 1/2
PI (4,5) P_2_	Phosphatidylinositol-4,5-bisphosphate
PLD	Phospholipase D
PM	Plasma membrane
PRRs	Pattern-recognizing receptors
*Pst* DC3000	*Pseudomonas syringae* pv. *tomato* DC3000
PTI	Pattern-triggered immunity
REM	Remorin
ROS	Reactive oxygen species
SA	Salicylic acid
VD	Verticillium dahliae
WT	Wild type
W/SRC	WASP family verprolin homologous/SCAR regulatory complex

## References

[1] Ngou B.P.M., Ahn H.-K., Ding P., Jones J.D.G. (2021). Mutual potentiation of plant immunity by cell-surface and intracellular receptors. Nature.

[2] Macho A.P., Zipfel C. (2014). Plant PRRs and the activation of innate immune signaling. Mol. Cell.

[3] Li B., Meng X., Shan L., He P. (2016). Transcriptional regulation of pattern-triggered immunity in plants. Cell Host Microbe.

[4] Cao L., Wang W., Zhang W., Staiger C.J. (2022). Lipid signaling requires ROS production to elicit actin cytoskeleton remodeling during plant innate immunity. Int. J. Mol. Sci..

[5] Yu T.Y., Sun M.K., Liang L.K. (2021). Receptors in the induction of the plant innate immunity. Mol. Plant Microbe Interact..

[6] Cui H., Tsuda K., Parker J.E. (2015). Effector-triggered immunity: From pathogen perception to robust defense. Annu. Rev. Plant Biol..

[7] He F., Chen H., Han R. (2020). The plant cytoskeleton and crosslinking factors. Cell Bio..

[8] Wang L., Qiu T., Yue J., Guo N., He Y., Han X., Wang Q., Jia P., Wang H., Li M. (2021). *Arabidopsis ADF1* is regulated by MYB73 and is involved in response to salt stress affecting actin filament organization. Plant Cell Physiol..

[9] Li J., Staiger C.J. (2018). Understanding cytoskeletal dynamics during the plant immune response. Annu. Rev. Phytopathol..

[10] Lian N., Wang X., Jing Y., Lin J. (2021). Regulation of cytoskeleton-associated protein activities: Linking cellular signals to plant cytoskeletal function. J. Integr. Plant Biol..

[11] Hardham A.R. (2013). Microtubules and biotic interactions. Plant J..

[12] Porter K., Day B. (2016). From filaments to function: The role of the plant actin cytoskeleton in pathogen perception, signaling and immunity. J. Integr. Plant Biol..

[13] Qin L., Liu L., Tu J., Yang G., Wang S., Quilichini T.D., Gao P., Wang H., Peng G., Blancaflor E.B. (2021). The ARP2/3 complex, acting cooperatively with Class I formins, modulates penetration resistance in *Arabidopsis* against powdery mildew invasion. Plant Cell.

[14] Leontovyčová H., Kalachova T., Janda M. (2020). Disrupted actin: A novel player in pathogen attack sensing?. New Phytol..

[15] Zou M., Guo M., Zhou Z., Wang B., Pan Q., Li J., Zhou J.-M., Li J. (2021). MPK3- and MPK6-mediated VLN3 phosphorylation regulates actin dynamics during stomatal immunity in *Arabidopsis*. Nat. Commun..

[16] Henty-Ridilla J.L., Shimono M., Li J., Chang J.H., Day B., Staiger C.J. (2013). The plant actin cytoskeleton responds to signals from microbe-associated molecular patterns. PLoS Pathog..

[17] Opalski K.S., Schultheiss H., Kogel K.H., Hückelhoven R. (2005). The receptor-like MLO protein and the RAC/ROP family G-protein RACB modulate actin reorganization in barley attacked by the biotrophic powdery mildew fungus *Blumeria graminis* f.sp*. hordei*. Plant J..

[18] Yang L., Qin L., Liu G., Peremyslov V.V., Dolja V.V., Wei Y. (2014). Myosins XI modulate host cellular responses and penetration resistance to fungal pathogens. Proc. Natl. Acad. Sci. USA.

[19] Takemoto D., Jones D.A., Hardham A.R. (2003). GFP-tagging of cell components reveals the dynamics of subcellular re-organization in response to infection of *Arabidopsis* by oomycete pathogens. Plant J..

[20] Li L., Zhu X.M., Zhang Y.R., Cai Y.Y., Wang J.Y., Liu M.Y., Wang J.Y., Bao J.D., Lin F.C. (2022). Research on the molecular interaction mechanism between plants and pathogenic fungi. Int. J. Mol. Sci..

[21] Takemoto D., Jones D.A., Hardham A.R. (2006). Re-organization of the cytoskeleton and endoplasmic reticulum in the *Arabidopsis pen1-1* mutant inoculated with the non-adapted powdery mildew pathogen, *Blumeria graminis* f. sp*. hordei*. Mol. Plant Pathol..

[22] Sassmann S., Rodrigues C., Milne S.W., Nenninger A., Allwood E., Littlejohn G.R., Talbot N.J., Soeller C., Davies B., Hussey P.J. (2018). An immune-responsive cytoskeletal-plasma membrane feedback loop in plants. Curr. Biol..

[23] Kobayashi Y., Yamada M., Kobayashi I., Kunoh H. (1997). Actin microfilaments are required for the expression of nonhost resistance in higher plants. Plant Cell Physiol..

[24] Kobayashi I., Hakuno H. (2003). Actin-related defense mechanism to reject penetration attempt by a non-pathogen is maintained in tobacco BY-2 cells. Planta.

[25] Miklis M., Consonni C., Bhat R.A., Lipka V., Schulze-Lefert P., Panstruga R. (2007). Barley MLO modulates actin-dependent and actin-independent antifungal defense pathways at the cell periphery. Plant Physiol..

[26] Chen Y., Dangol S., Wang J., Jwa N.-S. (2020). Focal accumulation of ROS can block *Pyricularia oryzae* effector BAS4-expression and prevent infection in rice. Int. J. Mol. Sci..

[27] Katagiri F., Thilmony R., He S.Y. (2002). The *Arabidopsis thaliana*-*Pseudomonas syringae* interaction. Arabidopsis Book.

[28] Lee J., Teitzel G.M., Munkvold K., Del Pozo O., Martin G.B., Michelmore R.W., Greenberg J.T. (2012). Type III secretion and effectors shape the survival and growthpattern of *Pseudomonas syringae* on leaf surfaces. Plant Physiol..

[29] Leontovyčová H., Kalachova T., Trdá L., Pospíchalová R., Lamparová L., Dobrev P.I., Malínská K., Burketová L., Valentová O., Janda M. (2019). Actin depolymerization is able to increase plant resistance against pathogens via activation of salicylic acid signalling pathway. Sci. Rep..

[30] Henty-Ridilla J.L., Li J., Day B., Staiger C.J. (2014). ACTIN DEPOLYMERIZING FACTOR4 regulates actin dynamics during innate immune signaling in *Arabidopsis*. Plant Cell.

[31] Li J., Henty-Ridilla J.L., Staiger B.H., Day B., Staiger C.J. (2015). Capping protein integrates multiple MAMP signalling pathways to modulate actin dynamics during plant innate immunity. Nat. Commun..

[32] Arnaud D., Hwang I. (2015). A Sophisticated network of signaling pathways regulates stomatal defenses to bacterial pathogens. Mol. Plant.

[33] Melotto M., Zhang L., Oblessuc P.R., He S.Y. (2017). Stomatal defense a decade later. Plant Physiol..

[34] Shimono M., Higaki T., Kaku H., Shibuya N., Hasezawa S., Day B. (2016). Quantitative evaluation of stomatal cytoskeletal patterns during the activation of immune signaling in *Arabidopsis thaliana*. PLoS ONE.

[35] Hoefle C., Huesmann C., Schultheiss H., Börnke F., Hensel G., Kumlehn J., Hückelhoven R. (2011). A Barley ROP GTPase ACTIVATING PROTEIN associates with microtubules and regulates entry of the barley powdery mildew fungus into leaf epidermal cells. Plant Cell.

[36] Leelarasamee N., Zhang L., Gleason C. (2018). The root-knot nematode effector MiPFN3 disrupts plant actin filaments and promotes parasitism. PLoS Pathog..

[37] de Almeida Engler J., Van Poucke K., Karimi M., De Groodt R., Gheysen G., Engler G., Gheysen G. (2004). Dynamic cytoskeleton rearrangements in giant cells and syncytia of nematode-infected roots. Plant J..

[38] Jelenska J., Kang Y., Greenberg J.T. (2014). Plant pathogenic bacteria target the actin microfilament network involved in the trafficking of disease defense components. Bioarchitecture.

[39] Kang Y., Jelenska J., Cecchini N.M., Li Y., Lee M.W., Kovar D.R., Greenberg J.T. (2014). HopW1 from *Pseudomonas syringae* disrupts the actin cytoskeleton to promote virulence in *Arabidopsis*. PLoS Pathog..

[40] Shimono M., Lu Y., Porter K., Kvitko B.H., Henty-Ridilla J., Creason A., He S.Y., Chang J.H., Staiger C.J., Day B. (2016). The *Pseudomonas syringae* type III effector HopG1 induces actin remodeling to promote symptom development and susceptibility during infection. Plant Physiol..

[41] Tian J., Han L., Feng Z., Wang G., Liu W., Ma Y., Yu Y., Kong Z. (2015). Orchestration of microtubules and the actin cytoskeleton in trichome cell shape determination by a plant-unique kinesin. eLife..

[42] Sun H., Zhu X., Li C., Ma Z., Han X., Luo Y., Yang L., Yu J., Miao Y. (2021). *Xanthomonas* effector XopR hijacks host actin cytoskeleton via complex coacervation. Nat. Commun..

[43] Park E., Nedo A., Caplan J.L., Dinesh-Kumar S.P. (2018). Plant-microbe interactions: Organelles and the cytoskeleton in action. New Phytol..

[44] Lee A.H., Hurley B., Felsensteiner C., Yea C., Ckurshumova W., Bartetzko V., Wang P.W., Quach V., Lewis J.D., Liu Y.C. (2012). A bacterial acetyltransferase destroys plant microtubule networks and blocks secretion. PLoS Pathog..

[45] Guo M., Kim P., Li G., Elowsky C.G., Alfano J.R. (2016). A bacterial effector co-opts calmodulin to target the plant microtubule network. Cell Host Microbe.

[46] Cheong M.S., Kirik A., Kim J.G., Frame K., Kirik V., Mudgett M.B. (2014). AvrBsT acetylates *Arabidopsis* ACIP1, a protein that associates with microtubules and is required for immunity. PLoS Pathog..

[47] Erickson J.L., Adlung N., Lampe C., Bonas U., Schattat M.H. (2018). The *Xanthomonas* effector XopL uncovers the role of microtubules in stromule extension and dynamics in *Nicotiana benthamiana*. Plant J..

[48] Deeks M.J., Hussey P.J., Davies B. (2002). Formins: Intermediates in signal-transduction cascades that affect cytoskeletal reorganization. Trends Plant Sci..

[49] Ma Z., Liu X., Nath S., Sun H., Tran T.M., Yang L., Mayor S., Miao Y. (2021). Formin nanoclustering-mediated actin assembly during plant flagellin and DSF signaling. Cell Rep..

[50] Ma Z., Sun Y., Zhu X., Yang L., Chen X., Miao Y. (2022). Membrane nanodomains modulate formin condensation for actin remodeling in *Arabidopsis* innate immune responses. Plant Cell.

[51] Goode B.L., Eck M.J. (2007). Mechanism and Function of formins in the control of actin assembly. Annu. Rev. Biochem..

[52] Romero S., Didry D., Larquet E., Boisset N., Pantaloni D., Carlier M.-F. (2007). How ATP hydrolysis controls filament assembly from profilin-actin. J. Biol. Chem..

[53] Sun H., Qiao Z., Chua K.P., Tursic A., Liu X., Gao Y.-G., Mu Y., Hou X., Miao Y. (2018). Profilin negatively regulates formin-mediated actin assembly to modulate PAMP-triggered plant immunity. Curr. Biol..

[54] Wang W., Sun Y., Han L., Su L., Xia G., Wang H. (2017). Overexpression of *GhPFN2* enhances protection against *Verticillium dahliae* invasion in cotton. Sci. China Life Sci..

[55] Sun G., Feng C., Guo J., Zhang A., Xu Y., Wang Y., Day B., Ma Q. (2019). The tomato Arp2/3 complex is required for resistance to the powdery mildew fungus *Oidium neolycopersici*. Plant Cell Environ..

[56] Badet T., Léger O., Barascud M., Voisin D., Sadon P., Vincent R., Le Ru A., Balagué C., Roby D., Raffaele S. (2019). Expression polymorphism at the *ARPC4* locus links the actin cytoskeleton with quantitative disease resistance to *Sclerotinia sclerotiorum* in *Arabidopsis thaliana*. New Phytol..

[57] Qi T., Wang J., Sun Q., Day B., Guo J., Ma Q. (2017). TaARPC3, Contributes to wheat resistance against the stripe rust fungus. Front. Plant Sci..

[58] Guo J., Peng H., Qi T., Xu S., Islam M.A., Day B., Ma Q., Kang Z., Guo J. (2022). TaARPC5 is required for wheat defense signaling in response to infection by the stripe rust fungus. Crop J..

[59] Li J., Cao L., Staiger C.J. (2017). Capping protein modulates actin remodeling in response to reactive oxygen species during plant innate immunity. Plant Physiol..

[60] Inada N. (2017). Plant actin depolymerizing factor: Actin microfilament disassembly and more. J. Plant Res..

[61] Henty J.L., Bledsoe S.W., Khurana P., Meagher R.B., Day B., Blanchoin L., Staiger C.J. (2011). *Arabidopsis* actin depolymerizing factor4 modulates the stochastic dynamic behavior of actin filaments in the cortical array of epidermal cells. Plant Cell.

[62] Zhang B., Hua Y., Wang J., Huo Y., Shimono M., Day B., Ma Q. (2017). *TaADF4*, an actin-depolymerizing factor from wheat, is required for resistance to the stripe rust pathogen *Puccinia striiformis* f. sp. *tritici*. Plant J..

[63] Fu Y., Duan X., Tang C., Li X., Voegele R.T., Wang X., Wei G., Kang Z. (2014). TaADF7, an actin-depolymerizing factor, contributes to wheat resistance against *Puccinia striiformis* f. sp*. tritici*. Plant J..

[64] Sun Y., Zhong M., Li Y., Zhang R., Su L., Xia G., Wang H. (2021). GhADF6-mediated actin reorganization is associated with defence against *Verticillium dahliae* infection in cotton. Mol. Plant Pathol..

[65] Tang C., Deng L., Chang D., Chen S., Wang X., Kang Z. (2016). TaADF3, an actin-depolymerizing factor, negatively modulates wheat resistance against *Puccinia striiformis*. Front. Plant Sci..

[66] Inada N., Higaki T., Hasezawa S. (2016). Nuclear function of subclass I actin-depolymerizing factor contributes to susceptibility in *Arabidopsis* to an adapted powdery mildew fungus. Plant Physiol..

[67] Tian M., Chaudhry F., Ruzicka D.R., Meagher R.B., Staiger C.J., Day B. (2009). *Arabidopsis* actin-depolymerizing factor AtADF4 mediates defense signal transduction triggered by the *Pseudomonas syringae* effector AvrPphB. Plant Physiol..

[68] Huang S., Qu X., Zhang R. (2015). Plant villins: Versatile actin regulatory proteins. J. Integr. Plant Biol..

[69] Khurana P., Henty J.L., Huang S., Staiger A.M., Blanchoin L., Staiger C.J. (2010). *Arabidopsis* VILLIN1 and VILLIN3 have overlapping and distinct activities in actin bundle formation and turnover. Plant Cell.

[70] Zhang H., Qu X., Bao C., Khurana P., Wang Q., Xie Y., Zheng Y., Chen N., Blanchoin L., Staiger C.J. (2010). *Arabidopsis* VILLIN5, an actin filament bundling and severing protein, is necessary for normal pollen tube growth. Plant Cell.

[71] Zhang Y., Xiao Y., Du F., Cao L., Dong H., Ren H. (2011). *Arabidopsis* VILLIN4 is involved in root hair growth through regulating actin organization in a Ca²⁺-dependent manner. New Phytol..

[72] Zipfel C., Kunze G., Chinchilla D., Caniard A., Jones J.D., Boller T., Felix G. (2006). Perception of the bacterial PAMP EF-Tu by the receptor EFR restricts *Agrobacterium*-mediated transformation. Cell.

[73] Gómez-Gómez L., Boller T. (2000). FLS2: An LRR receptor-like kinase involved in the perception of the bacterial elicitor flagellin in *Arabidopsis*. Mol. Cell.

[74] Miya A., Albert P., Shinya T., Desaki Y., Ichimura K., Shirasu K., Narusaka Y., Kawakami N., Kaku H., Shibuya N. (2007). CERK1, a LysM receptor kinase, is essential for chitin elicitor signaling in *Arabidopsis*. Proc. Natl. Acad. Sci. USA.

[75] Wan J., Tanaka K., Zhang X., Son G.H., Brechenmacher L., Nguyen T.H.N., Stacey G. (2012). LYK4, a lysin motif receptor-like kinase, is important for chitin signaling and plant innate immunity in *Arabidopsis*. Plant Physiol..

[76] Cao Y., Liang Y., Tanaka K., Nguyen C.T., Jedrzejczak R.P., Joachimiak A., Stacey G. (2014). The kinase LYK5 is a major chitin receptor in *Arabidopsis* and forms a chitin-induced complex with related kinase CERK1. eLife.

[77] Huang S., Gao L., Blanchoin L., Staiger C.J. (2006). Heterodimeric capping protein from *Arabidopsis* is regulated by phosphatidic acid. Mol. Biol. Cell.

[78] Li J., Henty-Ridilla J.L., Huang S., Wang X., Blanchoin L., Staiger C.J. (2012). Capping protein modulates the dynamic behavior of actin filaments in response to phosphatidic acid in *Arabidopsis*. Plant Cell.

[79] Torres M.A., Dangl J.L., Jones J.D.G. (2002). *Arabidopsis* gp91phox homologues AtrbohD and AtrbohF are required for accumulation of reactive oxygen intermediates in the plant defense response. Proc. Natl. Acad. Sci. USA.

[80] Torres M.A., Jones J.D.G., Dangl J.L. (2006). Reactive oxygen species signaling in response to pathogens. Plant Physiol..

[81] Bredow M., Monaghan J. (2019). Regulation of plant immune signaling by calcium-dependent protein kinases. Mol. Plant-Microbe Interact..

[82] Li J., Wang X., Qin T., Zhang Y., Liu X., Sun J., Zhou Y., Zhu L., Zhang Z., Yuan M. (2011). MDP25, A novel calcium regulatory protein, mediates hypocotyl cell elongation by destabilizing cortical microtubules in *Arabidopsis*. Plant Cell.

[83] Qin T., Liu X., Li J., Sun J., Song L., Mao T. (2014). *Arabidopsis* microtubule-destabilizing protein 25 functions in pollen tube growth by severing actin filaments. Plant Cell.

[84] Giovannoni M., Marti L., Ferrari S., Tanaka-Takada N., Maeshima M., Ott T., De Lorenzo G., Mattei B. (2021). The plasma membrane-associated Ca^2+^-binding protein, PCaP1, is required for oligogalacturonide and flagellin-induced priming and immunity. Plant Cell Environ..

[85] Porter K., Shimono M., Tian M., Day B. (2012). *Arabidopsis* Actin-Depolymerizing Factor-4 links pathogen perception, defense activation and transcription to cytoskeletal dynamics. PLoS Pathog..

[86] Lu Y.J., Li P., Shimono M., Corrion A., Higaki T., He S.Y., Day B. (2020). *Arabidopsis* calcium-dependent protein kinase 3 regulates actin cytoskeleton organization and immunity. Nat. Commun..

[87] Benschop J.J., Mohammed S., O’Flaherty M., Heck A.J.R., Slijper M., Menke F.L.H. (2007). Quantitative phosphoproteomics of early elicitor signaling in *Arabidopsis*. Mol. Cell. Proteomics.

[88] Huesmann C., Reiner T., Hoefle C., Preuss J., Jurca M.E., Domoki M., Fehér A., Hückelhoven R. (2012). Barley ROP binding kinase1 is involved in microtubule organization and in basal penetration resistance to the barley powdery mildew fungus. Plant Physiol..

[89] Jedelská T., Luhová L., Petřivalský M. (2021). Nitric oxide signalling in plant interactions with pathogenic fungi and oomycetes. J Exp. Bot..

[90] Shi F.M., Yao L.L., Pei B.L., Zhou Q., Li X.L., Li Y., Li Y.Z. (2009). Cortical microtubule as a sensor and target of nitric oxide signal during the defence responses to *Verticillium dahlia* toxins in *Arabidopsis*. Plant Cell Environ..

[91] Qi Y., Katagiri F. (2012). Membrane microdomain may be a platform for immune signaling. Plant Signal Behav..

[92] Yu M., Cui Y., Zhang X., Li R., Lin J. (2020). Organization and dynamics of functional plant membrane microdomains. Cell Mol. Life Sci..

[93] Szymanski W.G., Zauber H., Erban A., Gorka M., Wu X.N., Schulze W.X. (2015). Cytoskeletal components define protein location to membrane microdomains. Mol. Cell Proteomics.

[94] Lv X., Jing Y., Xiao J., Zhang Y., Zhu Y., Julian R., Lin J. (2017). Membrane microdomains and the cytoskeleton constrain AtHIR1 dynamics and facilitate the formation of an AtHIR1-associated immune complex. Plant J..

[95] Ali G.S., Prasad K.V., Day I., Reddy A.S. (2007). Ligand-dependent reduction in the membrane mobility of FLAGELLIN SENSITIVE2, an *arabidopsis* receptor-like kinase. Plant Cell Physiol..

[96] Cui Y., Li X., Yu M., Li R., Fan L., Zhu Y., Lin J. (2018). Sterols regulate endocytic pathways during flg22-induced defense responses in *Arabidopsis*. Development.

[97] McKenna J.F., Rolfe D.J., Webb S.E.D., Tolmie A.F., Botchway S.W., Martin-Fernandez M.L., Hawes C., Runions J. (2019). The cell wall regulates dynamics and size of plasma-membrane nanodomains in *Arabidopsis*. Proc. Natl. Acad. Sci. USA.

[98] Ortiz-Morea F.A., Savatin D.V., Dejonghe W., Kumar R., Luo Y., Adamowski M., Van den Begin J., Dressano K., de Oliveira G.P., Zhao X. (2016). Danger-associated peptide signaling in *Arabidopsis* requires clathrin. Proc. Natl. Acad. Sci. USA..

[99] Birtwistle M.R., Kholodenko B.N. (2009). Endocytosis and signalling: A meeting with mathematics. Mol. Oncol..

[100] Ryan J.M., Nebenführ A. (2018). Update on myosin motors: Molecular mechanisms and physiological functions. Plant Physiol..

[101] Beck M., Zhou J., Faulkner C., MacLean D., Robatzek S. (2012). Spatio-temporal cellular dynamics of the *Arabidopsis* flagellin receptor reveal activation status-dependent endosomal sorting. Plant Cell.

[102] Savage Z., Duggan C., Toufexi A., Pandey P., Liang Y., Segretin M.E., Yuen L.H., Gaboriau D.C.A., Leary A.Y., Tumtas Y. (2021). Chloroplasts alter their morphology and accumulate at the pathogen interface during infection by *Phytophthora infestans*. Plant J..

[103] Park E., Caplan J.L., Dinesh-Kumar S.P. (2018). Dynamic coordination of plastid morphological change by cytoskeleton for chloroplast-nucleus communication during plant immune responses. Plant Signal. Behav..

[104] Kumar A.S., Park E., Nedo A., Alqarni A., Ren L., Hoban K., Modla S., McDonald J.H., Kambhamettu C., Dinesh-Kumar S.P. (2018). Stromule extension along microtubules coordinated with actin-mediated anchoring guides perinuclear chloroplast movement during innate immunity. eLife.

[105] Li P., Day B. (2019). Battlefield cytoskeleton: Turning the tide on plant immunity. Mol. Plant-Microbe Interact..

[106] Škalamera D., Jibodhand S., Heath M.C. (1997). Callose deposition during the interaction between cowpea (*Vigna unguiculata*) and the monokaryotic stage of the cowpea rust fungus (*Uromyces vignae*). New Phytol..

[107] Underwood W., Somerville S.C. (2013). Perception of conserved pathogen elicitors at the plasma membrane leads to relocalization of the *Arabidopsis* PEN3 transporter. Proc. Natl. Acad. Sci. USA.

[108] Kusner D.J., Barton J.A., Qin C., Wang X., Iyer S.S. (2003). Evolutionary conservation of physical and functional interactions between phospholipase D and actin. Arch. Biochem. Biophys..

[109] Pleskot R., Potocky M., Pejchar P., Linek J., Bezvoda R., Martinec J., Valentová O., Novotná Z., Žárský V. (2010). Mutual regulation of plant phospholipase D and the actin cytoskeleton. Plant J..

[110] Matoušková J., Janda M., Fišer R., Šašek V., Kocourková D., Burketová L., Dušková J., Martinec J., Valentová O. (2014). Changes in actin dynamics are involved in salicylic acid signaling pathway. Plant Sci..

[111] Quentin M., Baurès I., Hoefle C., Caillaud M.-C., Allasia V., Panabières F., Abad P., Hückelhoven R., Keller H., Favery B. (2016). The *Arabidopsis* microtubule-associated protein MAP65-3 supports infection by filamentous biotrophic pathogens by down-regulating salicylic acid-dependent defenses. J. Exp. Bot..

[112] Qiao F., Chang X.-L., Nick P. (2010). The cytoskeleton enhances gene expression in the response to the Harpin elicitor in grapevine. J. Exp. Bot..

[113] Irieda H., Inoue Y., Mori M., Yamada K., Oshikawa Y., Saitoh H., Uemura A., Terauchi R., Kitakura S., Kosaka A. (2018). Conserved fungal effector suppresses PAMP-triggered immunity by targeting plant immune kinases. Proc. Natl. Acad. Sci. USA.

[114] Zhang X., Man Y., Zhuang X., Shen J., Zhang Y., Cui Y., Yu M., Xing J., Wang G., Lian N. (2021). Plant multiscale networks: Charting plant connectivity by multi-level analysis and imaging techniques. Sci. China Life Sci..

[115] Shen W., Ma L., Zhang X., Li X., Zhao Y., Jing Y., Feng Y., Tan X., Sun F., Lin J. (2020). Three-dimensional reconstruction of *Picea wilsonii* Mast. pollen grains using automated electron microscopy. Sci. China Life Sci..

[116] Garlick E., Faulkner E.L., Briddon S.J., Thomas S.G. (2022). Simple methods for quantifying super-resolved cortical actin. Sci. Rep..

[117] Vernet H., Fullana A.M., Sorribas F.J., Gualda E.J. (2022). Development of microscopic techniques for the visualization of plant–root-knot nematode interaction. Plants.

[118] Ovečka M., Sojka J., Tichá M., Komis G., Basheer J., Marchetti C., Šamajová O., Kuběnová L., Šamaj J. (2022). Imaging plant cells and organs with light-sheet and super-resolution microscopy. Plant Physiol..

